# Induction of Krüppel-Like Factor 4 Mediates Polymorphonuclear Neutrophil Activation in *Streptococcus pneumoniae* Infection

**DOI:** 10.3389/fmicb.2020.582070

**Published:** 2021-02-03

**Authors:** Aritra Bhattacharyya, Toni Herta, Claudia Conrad, Doris Frey, Pedro García, Norbert Suttorp, Stefan Hippenstiel, Janine Zahlten

**Affiliations:** ^1^Department of Infectious Diseases and Respiratory Medicine, Charité – Universitätsmedizin Berlin, Berlin, Germany; ^2^Departamento de Biotecnología Microbiana y de Plantas, Centro de Investigaciones Biológicas Margarita Salas, Consejo Superior de Investigaciones Científicas, Madrid, Spain; ^3^CIBER de Enfermedades Respiratorias, Madrid, Spain

**Keywords:** infectious diseases, innate immunity, neutrophils, Krüppel-like factor 4, *S. pneumoniae*

## Abstract

The recruitment and activation of polymorphonuclear neutrophils (PMNs) are of central importance for the elimination of pathogens in bacterial infections. We investigated the *Streptococcus pneumoniae*-dependent induction of the transcription factor Krüppel-like factor (KLF) 4 in PMNs as a potential regulator of PMN activation. We found that KLF4 expression is induced in human blood-derived PMNs in a time- and dose-dependent manner by wild-type *S. pneumoniae* and capsule knockout mutants. Unencapsulated knockout mutants induced stronger KLF4 expression than encapsulated wild types. The presence of autolysin LytA-competent (thus viable) pneumococci and LytA-mediated bacterial autolysis were required for KLF4 induction in human and murine PMNs. LyzMcre-mediated knockdown of KLF4 in murine blood-derived PMNs revealed that KLF4 influences pneumococci killing and increases the release of the proinflammatory cytokines tumor necrosis factor α and keratinocyte chemoattractant and decreases the release of the anti-inflammatory cytokine interleukin-10. Thus, *S. pneumoniae* induces KLF4 expression in PMNs, which contributes to PMN activation in *S. pneumoniae* infection.

## Introduction

*Streptococcus pneumoniae* is the main causative agent of community-acquired pneumonia and meningitis in children younger than 5 years and adults older than 65 years (van de Beek et al., 2006; Pletz et al., 2012; Prina et al., 2015). *S. pneumoniae* can give rise to a variety of local (e.g., sinusitis and otitis media) or invasive (e.g., pneumococcal sepsis) infections. Different serotypes are responsible for different organ manifestations (Orihuela et al., 2003). The emergence of multidrug-resistant *S. pneumoniae* strains has increased the mortality and morbidity associated with pneumococcal infections (Van Bambeke et al., 2007). Available pneumococcal vaccines such as the polysaccharide vaccine or the pneumococcal conjugate vaccine provide insufficient immunization (Feldman and Anderson, 2020). Taken together, *S. pneumoniae* is responsible for approximately 40,000 deaths per year in the United States alone (Brooks and Mias, 2018). It is thus of utmost importance to unveil the molecular mechanism underlying pneumococcal infections to develop new therapeutic strategies.

*Streptococcus pneumoniae* has various virulence factors that are responsible for its pathogenicity. The capsule forms the outermost layer and protects, among other things, against phagocytosis (Kadioglu et al., 2008). Anchored in the underlying cell wall, *S. pneumoniae* expresses several proteinergic virulence factors: of particular importance is the group of choline-binding proteins comprising the pneumococcal surface protein (Psp) A (prevents complement C3 binding) (Tu et al., 1999), the choline-binding protein (Cbp) A (mediates docking to epithelial cells) (Hammerschmidt et al., 1997), and the autolysins (mediate bacterial lysis, e.g., for proliferation or release of intracellular components) (López and García, 2004). Autolysin LytA is the main autolysin in *S. pneumoniae*. LytA functions as a murein hydrolase and releases upon cleavage of covalent cell wall bonds intracellular virulence factors (such as pneumolysin) and cell components [such as lipoteichoic acid (LTA), teichoic acid (TA), and bacterial DNA fragments] (Moscoso and Claverys, 2004; Mellroth et al., 2012). These pneumococcal components are recognized as pathogen-associated molecular patterns by pattern recognition receptors (PRRs) expressed in innate immune cells [e.g., macrophages and polymorphonuclear neutrophils (PMNs)] and other cell types (e.g., epithelial and endothelial cells) (Takeuchi and Akira, 2010). Among the various PRRs, the group of Toll-like receptors (TLRs) plays a major role in the recognition of *S. pneumoniae* (Koppe et al., 2012). Pneumococcal LTA and TA activate TLR2 (Schröder et al., 2003), whereas TLR4 probably recognizes pneumolysin (Malley et al., 2003) or, more likely, host cell danger-associated patterns after cell lysis by pneumolysin (Erridge, 2010), and TLR9 recognizes pneumococcal DNA (Hemmi et al., 2000). Upon activation of PRRs, regulation of transcription factors initiates the immune response.

Our group previously showed that recognition of *S. pneumoniae* induces the expression of the transcription factor Krüppel-like factor (KLF) 4 in human bronchial epithelial cells (Zahlten et al., 2013) and murine bone marrow (BM)-derived macrophages (Herta et al., 2018). In bronchial epithelial cells, KLF4 induction is mediated via TLR9 after recognition of pneumococcal DNA (released via LytA-dependent autolysis) and activates anti-inflammatory signaling pathways (Zahlten et al., 2013; Zahlten et al., 2015). In macrophages, KLF4 is induced upon direct contact with viable pneumococci and free (prokaryotic or eukaryotic) DNA, again involving LytA-dependent bacterial autolysis and DNA recognition via TLR9 (and a hitherto unknown DNA sensor). In contrast to epithelial cells, KLF4 activates proinflammatory signaling pathways in macrophages (Herta et al., 2018). PMNs form an important part of the innate immune system and may be referred to as spearheads of the host defense against bacterial infections. They are recruited to the site of infection and activated upon release of cytokines such as interleukin (IL)-8 and tumor necrosis factor α (TNF-α) (Suzuki et al., 1996; Craig et al., 2009; Vieira et al., 2009). After recognition of bacterial components by PRRs (Thomas and Schroder, 2013), PMNs release proinflammatory cytokines (thereby amplifying the immune response) and eliminate pathogens mainly by phagocytosis, spanning of neutrophil extracellular traps, and release of antimicrobial compounds such as reactive oxygen species (ROS) (Brinkmann et al., 2004; Mayadas et al., 2014). To date, only one study has shown that KLF4 regulates PMN activation after induction of expression by lipopolysaccharide (LPS) or the Gram-negative bacterium *Escherichia coli*. In this study, KLF4-deficient murine PMNs exhibited reduced production of proinflammatory cytokines and ROS, impaired degranulation, and impaired bacterial killing and clearance (Shen et al., 2017). Here, we examined the induction of KLF4 in human PMNs by the Gram-positive bacterium *S. pneumoniae*. Furthermore, we studied the function of KLF4 in murine PMNs after infection with *S. pneumoniae*.

## Materials and Methods

### Chemicals

CpG (ODN M362) was purchased from Invivogen (United States), MALP-2 from Alexis Biochemicals (United States), and LPS from *Salmonella minnesota* from Enzo Life Sciences GmbH (Germany). All chemicals used were of analytical grade and received from commercial sources.

### Bacterial Strains and Bacterial Products

For the present study, the *S. pneumoniae* serotype 2 wild-type strain D39, the unencapsulated D39 mutants D39Δcps and R6×, and the unencapsulated and autolysin LytA-deficient mutant R6×ΔlytA were used. For infection of the cells, the bacterial strains were plated on 5% sheep blood Columbia agar plates supplemented with kanamycin for the mutant strains. The plates were incubated overnight at 37°C with 5% CO_2_. The next day, single bacterial colonies were transferred to THY and allowed to grow until mid-log phase at 37°C with 5% CO_2_. The bacteria were centrifuged at 1,800 *g* for 10 min, and the pellets were resuspended in RPMI 1640 with 2% fetal calf serum and 1% glutamine to the required concentration for the infection of cells. For the isolation of the bacterial DNA pellets, samples were resuspended in TES, and lysozyme and mutanolysin were added and incubated at 37°C for 1 h for cell lysis. RNase was added for 15 min at 37°C, followed by a 30-min incubation with proteinase K at 30°C. Then, 10% N-lauroylsarcosine sodium salt (in 250 mM EDTA) was added for 1 h at 37°C. To precipitate the DNA, phenol and sodium acetate were added. The DNA pellet was dissolved in TE buffer after a washing step with ethanol.

### Isolation of Human Blood PMNs

Human PMNs used in this study were isolated from buffy coats obtained from the German Red Cross blood transfusion service Berlin (Germany) using an EasySep^TM^ direct human neutrophil isolation kit (Stemcell^TM^ Technologies) following the manufacturer’s instructions. The isolated cells were resuspended in RPMI 1640 (Gibco, United States) supplemented with 2% fetal bovine serum (GE Healthcare, United States) and 1% L-glutamine (Sigma–Aldrich, United States).

### Animals

C57BL/6 ERT-cre ^+ /–^/KLF4^loxP/loxP^ mice and C57BL/6 ERT-cre^–/–^/KFL4^*loxP/loxP*^ mice (a kind gift from Gary K. Owens, Department of Molecular Physiology and Biological Physics, University of Virginia, Charlottesville) and B6.129P2-Lyz2tm1(cre) Ifo mice (Charles River, United States) were mated to generate myeloid KLF4 knockout mice (C57BL/6 LyzMcre^+/+^/KLF4^loxP/loxP^ mice, referred to as KLF4^–/–^) and KLF4 control mice (C57BL/6 LyzMcre^–/–^/KLF4^loxP/loxP^ mice, referred to as KLF4^+/+^). Heterozygous myeloid KLF4 knockout mice (C57BL/6 LyzMcre^+/–^/KLF4^loxP/loxP^) are referred to as KLF4^+/–^.

### Isolation of Murine Bone Marrow PMNs and Murine Blood PMNs

To obtain BM-derived PMNs, mice were anesthetized with xylazine and (Rotexmedica, France) ketamine intraperitoneally and exsanguinated via the *vena cava caudalis*. Femurs and tibias were detached, and soft tissue was removed. BM was flushed out with sterile phosphate-buffered saline (PBS), BM-cells were isolated as described in Swamydas et al. (2015), and BM-PMNs were selected using the MACS mouse anti-Ly-6G Microbead Kit (Miltenyi Biotech, Germany) following the manufacturer’s instructions. Selected BM-PMNs were resuspended in RPMI 1640 supplemented with 2% fetal bovine serum and 1% L-glutamine. To obtain murine blood PMNs, blood was collected from the *vena cava caudalis* after injection of 50 μL of heparin. Before the PMNs were isolated, the composition of the cells was analyzed using the Scil Vet ABC^TM^ Hematology Analyzer. The main blood cell fractions were lymphocytes (70%), PMNs (10%), and monocytes (5%). The remaining 15% were platelets and other granulocytes. There were no differences between the blood cells found in control (KLF4^+/+^) and myeloid KLF4 knockout (KLF4^–/–^) mice (data not shown). Red blood cells (RBCs) were lyzed with RBC lysis buffer (155 mM NH_4_Cl, 10 mM KHCO_3_, 10 nM EDTA-Na; pH 7.4), and PMNs were selected using the MACS mouse anti-Ly-6G Microbead Kit. Blood PMNs and the remaining white blood cells (referred to as WBCΔPMNs) were separately resuspended in RPMI 1640 with 2% fetal bovine serum and 1% L-glutamine. To obtain enough PMNs for one experiment, the blood of 10–11 mice per group was pooled to obtain approximately 1 × 10^6^ blood-derived PMNs.

### Stimulation of Human and Murine PMNs

Human and murine PMNs were stimulated with *S. pneumoniae* bacterial suspensions [1 × 10^6^ colony-forming units (CFU)/mL or 1 × 10^8^ CFU/mL for multiplicity of infection (MOI) 1, 10, or 100] for 3 or 6 h, or 5 μg/mL isolated R6× DNA, 0.05 ng/μL MALP-2, 10 ng/μL CpG, or 0.1 ng/μL LPS for 6 h in RPMI 1640 supplemented with 2% fetal bovine serum and 1% L-glutamine for Western blot experiments. For enzyme-linked immunosorbent assay (ELISA) experiments, murine blood-derived PMNs were stimulated with D39 MOI 1 for 16 h. Supernatants were harvested, centrifuged, and subjected to analysis.

### Western Blot

Cells were lyzed in lysis buffer containing NP40. Forty micrograms of total protein was subjected to sodium dodecyl sulfate-polyacrylamide gel electrophoresis (using 10% gels) and transferred to Hybond-ECL membranes (GE Healthcare, United States). Membranes were blocked with Odyssey blocking buffer (LI-COR Biosciences, United States) for 2 h at room temperature and incubated with primary antibodies against KLF4 (catalog #sc-20691, Santa Cruz Biotechnology, United States) or β-actin (catalog #sc-130656, Santa Cruz Biotechnology, United States) each 1:1,000 overnight at 4°C. Membranes were washed with PBST (1 × PBS + 0.01% Tween-20) and incubated with the respective secondary antibodies anti-rabbit Cy5.5 (1:2,000) or anti-goat IRDye800 (1:2,000) purchased from Rockland (United States) for 1 h at room temperature. Protein levels were detected and quantified using a LI-COR Odyssey 2.0 infrared imaging system (LI-COR Biosciences, United States). The quantification of the KLF4 and β-actin bands was performed with strict regard to the methodical requirements as described in Taylor et al. (2013).

### CFU Assay

*Streptococcus pneumoniae* strains D39 and R6× were grown to midlogarithmic phase as described above. The bacterial pellet was resuspended in Hanks balanced salt solution (HBSS) with calcium and magnesium (Gibco, United States). Ten microliters of 1 × 10^8^ CFU/mL of the bacterial suspension was opsonized with 40 μL of serum from the respective mouse strain for 30 min at 37°C and added to the murine PMNs at a concentration of 1 × 10^7^ CFU/mL (MOI 100) for 1 or 3 h at 37°C. Lysis buffer (10% Triton X-100 in HBSS) was then supplemented into the medium and incubated for 10 min at 37°C. Serial dilutions were plated on Columbia agar plates containing 5% sheep blood (BD Biosciences, United States) and incubated overnight at 37°C. The next day, colonies were counted, and CFUs were calculated. The results were shown as CFU in percent of input (set to 100%).

### ELISA

Mouse TNF-α, keratinocyte chemoattractant (KC), IL-1β, and IL-10 in the supernatants of stimulated cells were measured using TNF-α (eBioscience^TM^, United States), KC, IL-1β, and IL-10 (R&D Systems, United States) ELISA kits following the manufacturer’s instructions.

### Data Analysis

Statistical analysis was performed using GraphPad Prism 6 (GraphPad, United States). The results were compared using the Kruskal–Wallis test [non-parametric one-way analysis of variance (ANOVA)] with Dunn multiple-comparisons test, non-parametric two-way ANOVA with Bonferroni *post hoc* test, or unpaired *t*-test as specified in the figure legends. *P* < 0.05 was considered statistically significant.

## Results

### KLF4 Is Induced in Human PMNs by *S. pneumoniae* and Requires the Presence of LytA-Competent Pneumococci

**FIGURE 1:**
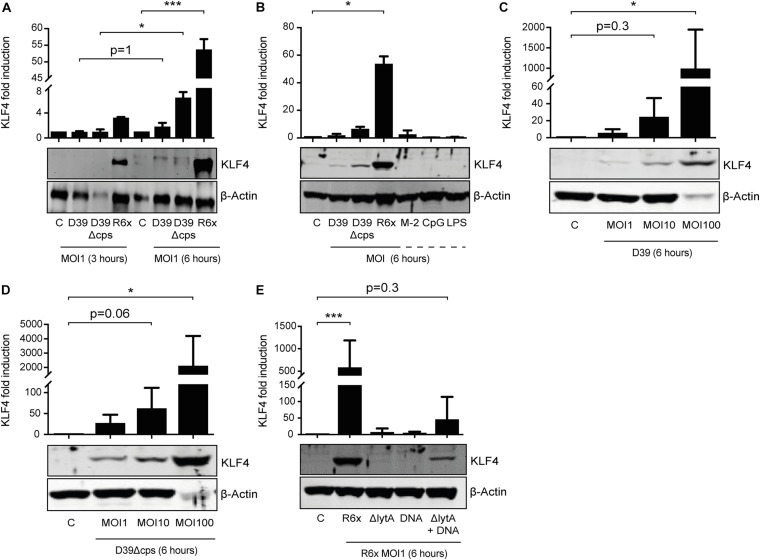
Induction of KLF4 expression in human blood-derived PMNs by *S. pneumoniae* requires LytA-dependent bacterial autolysis. PMNs isolated from human blood were stimulated with D39, D39Δcps, or R6× pneumococci with different MOIs (1, 10, or 100) for 3 or 6 h **(A–D)**, with 0.05 ng/μL MALP-2 (M-2), 10 ng/μL CpG, or 0.1 ng/μL LPS for 6 h **(B)**, or with R6×, R6×ΔlytA (MOI 1), or 5 μg/mL R6× DNA alone or in combination with R6×ΔlytA (MOI 1) **(E)**. Cell lysates were collected after stimulation and analyzed for KLF4 expression using Western blotting. β-Actin confirmed equal protein loading. The densitometries of the KLF4 and β-actin bands were quantified using an Odyssey 2.0 infrared imaging system. The ratios of the KLF4 and β-actin densitometries were calculated and shown as the fold change of induction to unstimulated PMNs (control, **C**). Quantifications show the mean with standard deviation of at least three **(A–D)** or four **(E)** independent experiments. Statistics: Two-way ANOVA with Bonferroni *post hoc* test **(A)**. Kruskal–Wallis test with Dunn multiple-comparisons test **(B–E)**. *p < 0.05; ***p < 0.001. Each experiment was performed with PMNs isolated from the buffy coat of a different donor.

We previously showed that *S. pneumoniae* induces KLF4 in murine macrophages via TLR9, the TLR adapter proteins MyD88 and TRIF, and a hitherto unknown host cell DNA sensor (Herta et al., 2018). Nothing is known about the *S. pneumoniae*-dependent induction of KLF4 in PMNs. Therefore, we stimulated PMNs isolated from human blood with wild-type *S. pneumoniae* and the two capsule-deficient mutants D39Δcps and R6× for 3 h (Figure 1A) or 6 h (Figures 1A–D) with different MOIs (MOI 1, Figures 1A–E and Supplementary Figure 1, or MOI 10 and MOI 100, Figures 1C,D). We found that unstimulated human PMNs did not express KLF4, whereas D39, D39Δcps, and R6× induced KLF4 expression. While R6× induced KLF4 already after 3 h of infection with an MOI of 1, higher MOIs (10 and 100) and longer infection times (6 h) were needed with D39 and D39Δcps. Cells were then stimulated with the TLR2 agonist MALP-2, the TLR4 agonist LPS, or the TLR9 agonist CpG for 6 h. However, no induction of KLF4 was detectable (Figure 1B). As pneumococcal DNA released by autolysin LytA-dependent autolysis is required for the induction of KLF4 in human lung epithelial cells (Zahlten et al., 2015) and murine macrophages (Herta et al., 2018), we stimulated human blood PMNs with R6×, the autolysin LytA-deficient R6× mutant R6×ΔlytA, or R6× DNA alone or in combination with R6×ΔlytA (MOI 1 for 6 h) (Figure 1E). While KLF4 was not induced by R6×ΔlytA and R6× DNA alone, the combination of R6×ΔlytA and R6× DNA partly restored KLF4 induction. We observed a similar induction mechanism in murine blood-derived PMNs (Supplementary Figure 1). Thus, *S. pneumoniae* induces KLF4 in human and murine PMNs. This induction is not exclusively mediated via TLR2, TLR4, or TLR9. It requires the presence of intact *S. pneumoniae* and the autolysin LytA-dependent release of intracellular bacterial components (e.g., bacterial DNA).

### KLF4 Knockout in Murine PMNs Influences Pneumococcal Killing

**FIGURE 2:**
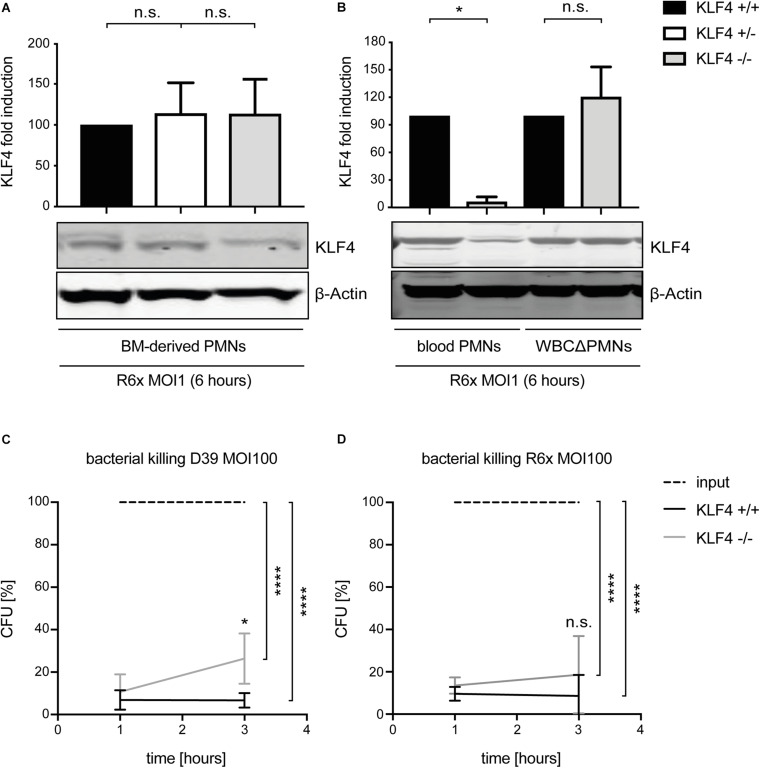
LyzMcre KLF4 knockout develops in mature but not premature murine PMNs and reduces the killing of *S. pneumoniae* in murine blood-derived PMNs. PMNs were isolated from bone marrow **(A)** or blood **(B)** of control (KLF4^+/+^, black bars), heterozygous myeloid KLF4 knockout (KLF^+/−^, white bars), and homozygous myeloid KLF4 knockout (KLF4^− /−^, gray bars) mice and stimulated with R6× pneumococci (MOI 1 for 6 h). Cell lysates were collected after stimulation and analyzed for KLF4 expression using Western blotting. β-Actin confirmed equal protein loading. The densitometries of the KLF4 and β-actin bands were quantified using an Odyssey 2.0 infrared imaging system. The ratios of the KLF4 and β-actin densitometries were calculated and are shown as the fold change of induction to control (KLF4^+/+^) PMNs. Quantifications show the mean with standard deviation of three independent experiments. Blood-derived PMNs from control (KLF4^+/+^, black lines) and KLF4 knockout (KLF4^− /−^, gray lines) mice were stimulated with opsonized D39 **(C)** or opsonized R6× pneumococci **(D)** (MOI 100 for 1 and 3 h, input, dashed lines) for the CFU assay. Graphs show mean with standard deviation of CFU in percent of input (set to 100%) of three independent experiments. Statistics: Kruskal–Wallis test with Dunn multiple-comparisons test **(A,B)** or two-way ANOVA with Bonferroni *post hoc* test **(C,D)**. ****p < 0.0001; *p < 0.05; n.s., not significant.

To study the function of KLF4 in murine PMNs, myeloid-specific KLF4 knockout mice with a C57BL/6 background were used. PMNs were isolated from the blood and BM of these animals and stimulated with R6× (MOI 1 for 6 h) to assess KLF4 knockout efficacy. While KLF4 induction by R6× did not differ in BM-derived PMNs (Figure 2A) and other remaining white blood cells after PMN isolation (WBCΔPMNs, Figure 2B) obtained from control (KLF4^+/+^) and myeloid KLF4 knockout (KLF4^–/–^) mice, we found a strong (>80%) reduction in KLF4 expression in blood KLF4^–/–^ PMNs (Figure 2B) after stimulation with R6×. Thus, KLF4 knockout was developed only in mature but not premature myeloid PMNs in LyzMcre mice.

Given that bacterial killing is an important function of PMNs in innate immunity, we tested whether KLF4 deficiency in PMNs alters pneumococcal killing properties. Blood-derived PMNs from control (KLF4^+/+^) and myeloid KLF4 knockout (KLF4^–/–^) mice were incubated for 1 and 3 h with opsonized *S. pneumoniae* D39 (MOI 100) (Figure 2C) or R6× (MOI 100) (Figure 2D) to perform CFU assays. KLF4^+/+^ PMNs showed natural killing activity of ∼90–95% (which corresponds to ∼1.5 log scales) for both bacterial strains. KLF4^–/–^ PMNs showed a significant lower killing of D39 and a tendency toward reduced killing of R6× pneumococci compared to KLF4^+/+^ PMNs after 3 h of incubation, although differences between KLF4^+/+^ and KLF4^–/–^ PMNs did not exceed 1 log scale for both bacterial strains. Thus, the loss of KLF4 reduces pneumococci killing in PMNs.

### KLF4 Knockout in Murine PMNs Leads to Reduced Secretion of TNF-α and KC and Increased Secretion of IL-10 When Stimulated With *S. pneumoniae*

**FIGURE 3:**
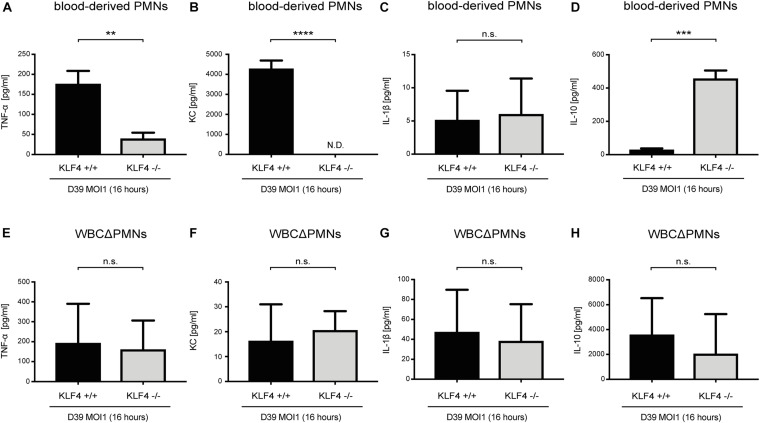
Knockout of KLF4 decreases the pneumococci-induced proinflammatory and increases the anti-inflammatory cytokine response in murine blood-derived PMNs. Blood PMNs and other white blood cells after PMN isolation (WBCΔPMNs) derived from control (KLF4^+/+^, black bars) and myeloid KLF4 knockout (KLF4^− /−^, gray bars) mice were stimulated with D39 pneumococci (MOI 1 for 16 h). Supernatants were collected, and TNF-α **(A,E)**, KC **(B,F)**, IL-1β **(C,G)**, and IL-10. **(D,H)** ELISAs were performed. Graphs show the mean with standard deviation of three independent experiments. Statistics: unpaired *t*-test. **p < 0.01; ***p < 0.001; ****p < 0.0001; n.s., not significant; N.D., not detectable.

Cytokines such as TNF-α, KC, IL-1β, and IL-10, released, among others, by PMNs, orchestrate the innate immune response in bacterial infections (Borish and Steinke, 2003; Turner et al., 2014). We previously showed that knockout of KLF4 in murine macrophages reduces the release of proinflammatory cytokines and increases the release of the anti-inflammatory cytokine IL-10 after stimulation with *S. pneumoniae* (Herta et al., 2018). To investigate whether KLF4 modulates pneumococci-dependent proinflammatory and anti-inflammatory cytokine release in murine PMNs, we stimulated blood-derived PMNs and other WBCΔPMNs from control (KLF4^+/+^) and myeloid KLF4 knockout (KLF4^–/–^) mice with *S. pneumoniae* D39 (MOI 1 for 16 h) and measured proinflammatory TNF-α, KC, and IL-1β as well as anti-inflammatory IL-10 cytokine release using ELISAs. The release of TNF-α and KC was significantly reduced, and the release of IL-10 increased in KLF4^–/–^ blood-derived PMNs compared to the respective control cells (Figures 3A,B,D), whereas WBCΔPMNs showed no difference in TNF-α, KC, and IL-10 release (Figures 3E,F,H). The release of IL-1β was unaffected in PMNs and WBCΔPMNs (Figures 3C,G). Thus, KLF4 increases proinflammatory TNF-α and KC but not IL-1β release and decreases anti-inflammatory IL-10 release in murine PMNs when stimulated with *S. pneumoniae*.

## Discussion

In the present study, we identified the transcription factor KLF4 as a potential mediator of PMN activation in *S. pneumoniae* infection. The following observations support this finding: (i) while KLF4 was not detectable in unstimulated murine and human PMNs, stimulation with *S. pneumoniae* induced the expression of KLF4. Interestingly, the induction mechanism in PMNs is partly similar to the one we described in macrophages (Herta et al., 2018) and epithelial cells (Zahlten et al., 2013, 2015); in all cell types, autolysin LytA-mediated bacterial autolysis was necessary to induce KLF4. The activation of single TLRs by TLR agonists (MALP-2, LPS, and CpG) was not sufficient to foster KLF4 expression, neither in macrophages nor in epithelial cells or PMNs. In macrophages and PMNs, the abolished or reduced induction of KLF4 expression by LytA-deficient pneumococcal mutants could at least partly be restored by the addition of free (bacterial) DNA. In both cell types, encapsulated pneumococci induced a weaker KLF4 expression, which might indicate that (in addition to free bacterial DNA) the recognition of a so far unidentified pneumococcal cell wall component (partly covered by the capsule) is necessary to induce KLF4. As macrophages and PMNs arise from common myeloid precursors, both cell types share several characteristics, such as similar transcriptional profiles and overlapping expression of PRRs (Silva, 2010; Prame Kumar et al., 2018). Thus, a similar induction mechanism of KLF4 by *S. pneumoniae* in both cell types is in line with these observations. Shen et al. (2017) support our finding, as they reported a strong induction of KLF4 in murine BM-derived PMNs when stimulated with *E. coli*. However, they also found an induction of KLF4 after stimulation with the TLR4-ligand LPS. A possible explanation for this conflicting result might be the use of human (in this study) or murine PMNs (Shen et al., 2017) for LPS stimulation. TLR4’s function with respect to intracellular regulation of signaling pathways upon stimulation with LPS may vary between different species (Vaure and Liu, 2014). Moreover, the affinity and sensitivity of TLR4 for its ligand LPS are different in mice and humans, as the ligand-binding domain exhibits considerable sequence divergence (Werling et al., 2009). This is potentially important as LPS from different sources was used (*S. minnesota* versus *E. coli*), and the induction of KLF4 was assessed with techniques that differ in sensitivity and kinetics of regulation (protein level by Western blotting versus mRNA level by quantitative PCR). Neutrophils are relatively non-responsive to a single stimulus, but exposure to one stimulus (e.g., LPS) enhances the ability to mount a strong activation in response to a second stimulus (referred to as PMN priming) (Swain et al., 2002; Mayadas et al., 2014). As KLF4 is a potential mediator of PMN activation (discussed below), the necessity of more than one stimulus to induce KLF4 expression (as we observed with *S. pneumoniae*) is explainable. Overall, KLF4 is induced in PMNs in response to pathogen stimulation (*S. pneumoniae*, *E. coli*). (ii) KLF4 expression is involved in PMN activation in *S. pneumoniae* infection by increasing the release of proinflammatory cytokines and reducing the release of the anti-inflammatory cytokine IL-10. To study the function of KLF4 in PMNs, the LyzMcre system was applied to generate myeloid-specific KLF4 knockout mice. In line with Clausen et al. (1999), we found that knockout of KLF4 was developed in mature (blood-derived) but not premature (BM-derived) PMNs or in the remainder of the WBC fraction after PMN isolation in our system. Thus, mature blood-derived PMNs from control (KLF4^+/+^) and myeloid KLF4 knockout (KLF4^–/–^) mice were used to assess KLF4 function. Again, similar to macrophages (Herta et al., 2018), knockdown of KLF4 in murine blood-derived PMNs strongly reduced the release of the proinflammatory cytokines TNF-α and KC (the murine homolog of human IL-8) and increased the release of IL-10 in response to *S. pneumoniae* stimulation. Shen et al. (2017) support this finding, as they observed blunted transcription of TNF-α in KLF4-deficient murine PMNs. The release of the proinflammatory cytokine IL-1β was not affected by KLF4 knockout in murine PMNs. As the production of IL-1β is regulated by inflammasomes [while TNF-α and KC strongly depend on nuclear factor κB (NF-κB)] (Liu et al., 2017; Chan and Schroder, 2020), KLF4 might influence the release of NF-κB-dependent but not inflammasome-dependent proinflammatory cytokines. As expected, we could not observe any changes in the cytokine response in pneumococci-stimulated WBCsΔPMNs. The main fraction of these cells is lymphocytes that are not affected by the LyzMcre-mediated knockdown of KLF4 (Clausen et al., 1999).

Because of their robust reactivity with potential host tissue-damaging activity (Nathan, 2006), PMNs are typically not resident in tissue and organs. Instead, they circulate as quiescent cells in the blood, are ready to become recruited to the site of infection, and are activated by cytokines (Prame Kumar et al., 2018). The importance of TNF-α and KC/IL-8 for PMN recruitment and activation is well known (Vieira et al., 2009; Mayadas et al., 2014). The source of these cytokines was believed to be tissue resident immune and non-immune cells. However, the view of PMNs as terminally differentiated effectors solely regulated by external signals is changing (Mayadas et al., 2014). PMNs, upon activation, produce a variety of mediators and cytokines (including TNF-α, KC/IL-8, and IL-10) with regulatory effects on the immune response, including their own activation and recruitment (Sadik et al., 2011; Tecchio et al., 2014). Thus, we reason that KLF4 (among other transcription factors) mediates PMN activation in *S. pneumoniae* infection by increasing the release of TNF-α and KC/IL-8 and reducing the release of IL-10.

In line with Shen et al. (2017), KLF4 might influence pneumococci killing in PMNs. However, in contrast to previous results with *E. coli* (Shen et al., 2017), pneumococci killing activity was reduced by less than 1 log scale after knockdown of KLF4. PMNs dispose of several intracellular and extracellular mechanisms for bacterial killing, dependent on oxygen and its reactive intermediates (e.g., ROS) or oxygen-independent mechanisms (e.g., antimicrobial peptides, granule proteases, lysozymes) (Hampton et al., 1998; Nauseef, 2007). Pathogens differ in their sensitivity to these mechanisms. *S. pneumoniae* successfully evades the respiratory burst response in PMNs (Schaper et al., 2003; Marriott et al., 2008) but is sensitive to granule proteases (Standish and Weiser, 2009), whereas *E. coli* is strongly targeted by ROS (Phan et al., 2018). KLF4 might thus influence oxygen-dependent but to a lesser extent oxygen-independent bacterial killing mechanisms in PMNs.

In summary, the obtained results underline the importance of the transcription factor KLF4 as a regulator of the innate immune response in *S. pneumoniae* infection. While KLF4 in PMNs and macrophages (Herta et al., 2018) activates the release of proinflammatory and inhibits the release of anti-inflammatory cytokines, it counteracts this effect in epithelial cells by inhibiting the release of proinflammatory and activating the release of anti-inflammatory cytokines (Zahlten et al., 2013, 2015). We therefore speculate that KLF4 induction in myeloid cells promotes *S. pneumoniae* elimination, whereas KLF4 induction in epithelial cells acts as an anti-inflammatory safety mechanism to prevent hyperinflammation and host tissue destruction in *S. pneumoniae* infection. The present study, together with our previous findings in macrophages and epithelial cells, provides a rationale to further investigate the role of KLF4 in *S. pneumoniae* infection *in vivo*.

## Data Availability Statement

The raw data supporting the conclusions of this article will be made available by the authors, without undue reservation.

## Ethics Statement

Animal housing and experimental procedures were complied with the Federation of European Laboratory Animal Science Associations (FELASA) guidelines and recommendations for the care and use of laboratory animals. The animal procedures were approved by the local institutional (Charité – Universitätsmedizin Berlin) and governmental [Landesamt für Gesundheit und Soziales Berlin (LAGeSo), approval ID: T0087/15] authorities. Permission for experiments with human primary cells was obtained from Charité Ethics Committee (Charité – Universitätsmedizin Berlin). All experimental procedures (working with genetically modified organisms, biological agents, and chemicals) were performed in accordance with the local institutional (Charité–Universitätsmedizin Berlin) and governmental [Landesamt für Gesundheit und Soziales Berlin (LAGeSO) and Landesamt für Arbeitsschutz, Gesundheitsschutz und technische Sicherheit Berlin (LAGetSi)] authorities.

## Author Contributions

JZ conceived the experiments. AB, CC, and DF performed the experiments. AB, JZ, and SH analyzed the data. PG provided the DNA template to generate the LytA-deficient R6× strain. JZ, NS, and SH supervised the project. TH, AB, and JZ wrote the manuscript. AB and TH prepared the figures. All authors reviewed and edited the manuscript.

## Conflict of Interest

The authors declare that the research was conducted in the absence of any commercial or financial relationships that could be construed as a potential conflict of interest.
