# Supplementary material for: Induction of Krüppel-Like Factor 4 Mediates Polymorphonuclear Neutrophil Activation in Streptococcus pneumoniae Infection
Source: Front Microbiol. 2021 Feb 3;11:582070. doi: 10.3389/fmicb.2020.582070 (PMC7887292; doi:10.3389/fmicb.2020.582070)
Supplement: Supplementary file 1 [file Image_1.pdf]

Induction of Krueppel-like factor 4 mediates polymorphonuclear neutrophil activation in *Streptococcus pneumoniae* infection

Aritra Bhattacharyya, Toni Herta, Claudia Conrad, Doris Frey, Pedro García, Norbert Suttorp, Stefan Hippenstiel and Janine Zahlten

Table of content

Supplementary Figure S1……...…………………………………………………………………...2

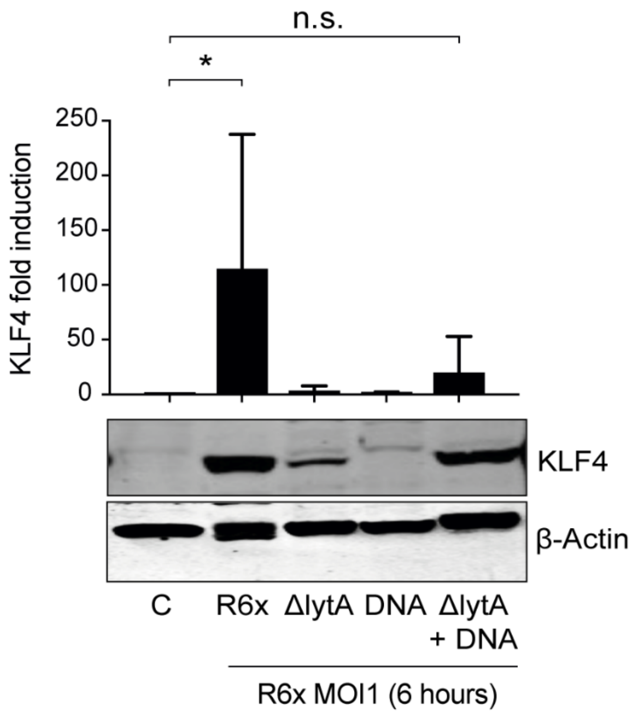

Supplementary Figure S1: Induction of KLF4 expression in murine blood-derived PMNs by *S. pneumoniae* requires LytA-dependent autolysis. PMNs were isolated from blood of control (KLF4$^{+/+}$) mice and stimulated with R6x, R6xΔlytA (MOI 1) or 5 µg/ml R6x DNA alone or in combination with R6xΔlytA (MOI 1). Cell lysates were collected after stimulation and analysed for KLF4 expression using western blotting. β-actin confirmed equal protein load. Densitometries of KLF4 and β-actin bands were quantified using Odyssey 2.0 infrared imaging system. Ratios of KLF4 and β-actin densitometries were calculated and shown as fold of induction to unstimulated PMNs (control, C). Quantifications show the mean with standard deviation of 3 independent experiments. Statistics: Kruskal-Wallis test with Dunn's multiple comparison test. * $p<0.05$; n.s., not significant.
